# Dermal fibroblasts cultured from donors with type 2 diabetes mellitus retain an epigenetic memory associated with poor wound healing responses

**DOI:** 10.1038/s41598-020-80072-z

**Published:** 2021-01-14

**Authors:** Aaiad H. A. Al-Rikabi, Desmond J. Tobin, Kirsten Riches-Suman, M. Julie Thornton

**Affiliations:** 1 Centre for Skin Sciences, Faculty of Life Sciences, University of Bradford, Bradford, UK; 2 The Charles Institute for Dermatology, School of Medicine, University College Dublin, Dublin, Ireland; 3 Department of Biology, College of Science, University of Baghdad, Baghdad, Iraq

**Keywords:** Cell biology, Biomarkers

## Abstract

The prevalence of Type 2 diabetes mellitus (T2DM) is escalating globally. Patients suffer from multiple complications including the development of chronic wounds that can lead to amputation. These wounds are characterised by an inflammatory environment including elevated tumour necrosis factor alpha (TNF-α). Dermal fibroblasts (DF) are critical for effective wound healing, so we sought to establish whether there were any differences in DF cultured from T2DM donors or those without diabetes (ND-DF). ND- and T2DM-DF when cultured similarly in vitro secreted comparable concentrations of TNF-α. Functionally, pre-treatment with TNF-α reduced the proliferation of ND-DF and transiently altered ND-DF morphology; however, T2DM-DF were resistant to these TNF-α induced changes. In contrast, TNF-α inhibited ND- and T2DM-DF migration and matrix metalloprotease expression to the same degree, although T2DM-DF expressed significantly higher levels of tissue inhibitor of metalloproteases (TIMP)-2. Finally, TNF-α significantly increased the secretion of pro-inflammatory cytokines (including CCL2, CXCL1 and SERPINE1) in ND-DF, whilst this effect in T2DM-DF was blunted, presumably due to the tendency to higher baseline pro-inflammatory cytokine expression observed in this cell type. Collectively, these data demonstrate that T2DM-DF exhibit a selective loss of responsiveness to TNF-α, particularly regarding proliferative and secretory functions. This highlights important phenotypic changes in T2DM-DF that may explain the susceptibility to chronic wounds in these patients.

## Introduction

Patients with type 2 diabetes mellitus (T2DM) suffer from impaired wound healing with persistent inflammation, micro- and macro-circulatory dysfunction, hypoxia, and impaired neuropeptide signalling. This can result in a chronic, non-healing, diabetic foot ulcer, with significant risk of foot, or lower limb amputation^1^. Typically, cutaneous wound healing follows three sequential, overlapping phases; an inflammatory phase, a proliferative phase and a remodelling phase^2^. The inflammatory phase takes place early in the sequence, lasting only a few days, followed by progression to tissue regeneration and re-epithelialisation, granulation tissue formation, angiogenesis and tissue remodelling^3^.

Dermal fibroblasts (DF) play a pivotal role during cutaneous wound healing; they proliferate and migrate into the wound bed to produce extracellular matrix (ECM) proteins that are essential for the generation of the granulation tissue and in providing a scaffold for the migration of inflammatory cells^2,4^. During the inflammatory phase DF also secrete an array of cytokines, chemokines and growth factors that synchronise the migration of immune cells to the wound bed and control their retention and survival in damaged tissue^5,6^. Hence, DF have a potential role in regulating the switch from acute inflammatory responses to chronic persistent inflammation^7^.

Chronic, non-healing wounds associated with diabetes exhibit an interruption in the normal healing process^8,9^. These wounds are characterised by a prolonged inflammatory phase, altered production of pro- and anti-inflammatory cytokines and impaired angiogenesis. DF exhibit diminished ECM synthesis and a decrease in their migratory and proliferative capacity. In addition, the migration and proliferation of keratinocytes is also decreased, which compromises the restoration of the epidermal barrier^10,11^.

The pro-inflammatory cytokine tumour necrosis factor alpha (TNF-α) plays a key role in controlling the inflammatory process during wound healing. However, continual, elevated levels of TNF-α can extend the inflammatory phase, which correlates with chronic, non-healing wounds, where there is no resolution of inflammation^12–14^. Therefore, the effects of TNF-α during the wound healing process are both time and concentration-dependent and TNF-α can initiate various effects on the DF. Lower concentrations of TNF-α promote remodelling/healing of injured tissue by stimulating inflammatory macrophages that secrete growth factors required to stimulate DF proliferation^15^. However, high concentrations of TNF-α for extended periods actually induce DF apoptosis with a deleterious effect on wound healing^16^. TNF-α inhibits their differentiation into myofibroblasts, which are important in the physical contraction of the wound^17^. High levels of TNF-α also inhibit synthesis of key ECM proteins such as fibronectin and type I collagen. Therefore, excessive and extensive exposure may contribute to chronic non-healing wounds prevalent in T2DM. Circulating plasma levels of TNF-α are typically around 10 pg/ml^18^ but in diabetic patients can be almost tenfold higher^19^ and circulating TNF-α concentrations positively correlate with diabetes duration^20^. In healthy skin tissue, concentrations of TNF-α are highly variable but typically in the order of 1.5 ng/ml^21^. However, TNF-α levels are three times higher in wound fluid from non-healing venous leg ulcers compared to healing ulcers^22^, and wounds of diabetic mice have higher levels of TNF-α compared to their normoglycaemic littermates^23^. Altogether, this implicates TNF-α in impaired wound healing in T2DM.

TNF-α also stimulates the secretion of proteolytic enzymes such as matrix metalloproteases (MMPs), specifically MMP-1,-2,-3,-9 and -13; elevated levels of which have been associated with chronic wounds^24–26^. In normal human skin, MMP expression is low and is controlled by tissue inhibitors of MMPs (TIMPs). However, during wound repair MMP secretion is stimulated by pro-inflammatory cytokines, which also down-regulate TIMP synthesis^27,28^. MMP secretion is required for wound repair because they have a key role in the degradation and remodelling of the ECM, in addition to facilitating the migration of keratinocytes and fibroblasts. However, failure to control their expression due to persistent, chronic inflammation, can lead to disproportionate proteolytic activity and excessive ECM degradation, resulting in an impaired healing response^28^. DF within these wounds are unable to proliferate and/or migrate since their functional receptors that respond to cytokines and growth factors have been down-regulated^26,28,29^.

While the role of DF in ECM assembly has long been recognised, additional roles during cutaneous wound healing are starting to emerge. Although inflammatory cells are the main source of TNF-α, it is also secreted by other by cells including DF^24,30^. Since DF have a central role in regulating acute and chronic inflammation, fibrosis, and the resolution of inflammation, they may act as a gatekeeper of the inflammatory response in human skin during wound healing.

In this study we sought to clarify whether there were phenotypic differences in primary DF cultures from the skin of donors with T2DM (T2DM-DF) and no diagnosis of diabetes (ND-DF), which may impact on their responses in a wound healing environment. Furthermore, their responses to TNF-α, which is prevalent in chronic wounds, was also assessed. While the basal secretion of TNF-α levels by T2DM-DF and ND-DF was similar, we discovered that T2DM-DF were resistant to the anti-proliferative, pro-quiescent and pro-inflammatory effects of TNF-α. By contrast, the anti-migratory stimulus of TNF-α was retained. Understanding the disparity in the function of T2DM-DF may help to identify markers for new therapeutic targets to improve the treatment of chronic, non-healing wounds and diabetic foot ulcers.

## Results

### Levels of TNF-α secreted by dermal fibroblasts in vitro are not affected by diabetes

**Figure 1:**
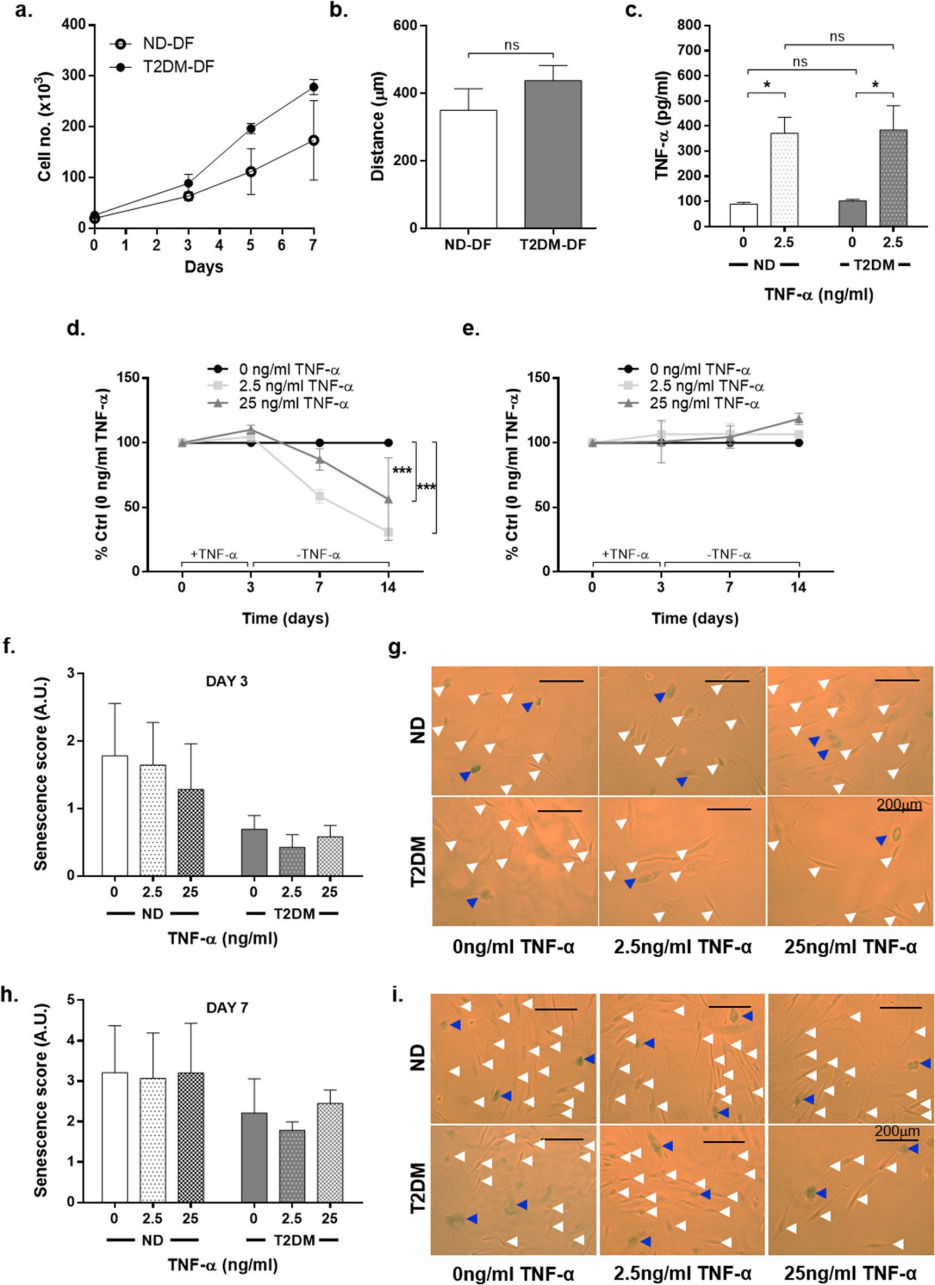
TNF-α has a differential effect on ND-DF and T2DM-DF proliferation. DF were quiesced for 24 h and then treated with DMEM + 10% FBS. (**a**) Basal proliferation rates were quantified by cell counting at days 2, 3 and 7 (n = 3 donors ND-DF, n = 4 donors T2DM-DF). (**b**) Basal migration rates were quantified using scratch wound assay (both n = 4 donors). (**c**) Confluent cells were quiesced for 24 h before being stimulated ± 2.5 ng/ml TNF-α for 24 h. Supernatants were collected and the concentration of TNF-α quantified (both n = 4 donors). (**d**) Cells were quiesced for 24 h and treated with 2.5 and 25 ng/ml TNF-α for 72 h before being transferred into full growth medium in the absence of TNF-α. Fluorescence was measured on days 0, 3, 7 and 14 and the percentage inhibition from control calculated for ND-DF and (**e**) T2DM-DF (both n = 4 donors). (**f**–**g**) Parallel cultures were fixed and stained for senescence-associated β-galactosidase in ND-DF and T2DM-DF at day 3 and (**h**–**i**) day 7 (both n = 4 donors). Blue arrowheads indicate senescent cells and white arrowheads indicate unstained cells. Two-way ANOVA with Sidak post-hoc test *P < 0.05, ***P < 0.001, ns = non-significant.

To establish whether there were any basal differences in the phenotype of ND-DF and T2DM-DF, we monitored their proliferation, migration and secretion of TNF-α under standard cell culture conditions. ND-DF increased in cell number by 1.7-fold in response to 10% FBS over 7 days. This was paralleled by an increase of 2.7-fold in T2DM-DF (ND-DF n = 3, T2DM-DF n = 3; Fig. 1a). Similarly, the migration rates of ND-DF and T2DM-DF over 24 h were comparable (350 μm vs 438 μm respectively; both n = 4; Fig. 1b). Furthermore, under non-stimulated conditions, ND-DF secreted 89.2 ± 6.8 pg/ml TNF-α, whereas T2DM-DF secreted 101.6 ± 5.9 pg/ml TNF-α (both n = 4, *P* = 0.8426, two-way ANOVA). As expected, in both populations there was significantly more TNF-α present in the conditioned medium (CM) following TNF-α treatment (*P* = 0.0023 for effect of TNF-α, two-way ANOVA), but this increase was the same regardless of diabetes status (371.5 ± 63.6 vs 384.2 ± 97.3 pg/ml respectively; two-way ANOVA, n = 4; Fig. 1c). Altogether, this demonstrates that the phenotype of DF under control conditions is not affected by T2DM, and that TNF-α secretion under basal and stimulated conditions is comparable between ND and T2DM-DF. However, we were interested in how the cells reacted to the pro-inflammatory stimulus of TNF-α to interrogate how wound healing is impacted in the hyper-inflammatory condition of T2DM.

### T2DM-DF are resistant to the anti-proliferative effects of TNF-α

Prior exposure to 2.5 ng/ml TNF-α for 3 days significantly inhibited ND-DF proliferation at 4 and 11-days post-TNF-α withdrawal by 41.3% and 69.1% respectively, despite the presence of 10% FBS (*P* = 0.0142 for effect of TNF-α, two-way ANOVA, n = 4). The higher concentration of 25 ng/ml TNF-α also significantly inhibited ND-DF proliferation but this was blunted and only significant at day 14 (by 43.7%; Fig. 1d), suggesting that 2.5 ng/ml was a maximal stimulus for proliferation. In contrast, TNF-α did not have any impact on T2DM-DF at either concentrations or time points tested (n = 4, Fig. 1e). The reduction in proliferation in ND-DF was not due to an increase in apoptosis as TNF-α had no impact on cell viability (n = 2).

### TNF-α does not induce dermal fibroblast senescence

We measured senescence-associated β-galacotosidase to investigate whether the reduced cell number in TNF-α-treated ND-DF primary cell cultures was due to induction of senescence. Three days after withdrawal of TNF-α, there was no significant difference between the senescence rates of ND-DF and T2DM-DF (1.78 ± 0.8 vs 0.69 ± 0.2 A.U., *P* = 0.1832, two-way ANOVA, n = 4). TNF-α concentration had no impact on senescence in either ND-DF or T2DM-DF (*P* = 0.1556, two-way ANOVA, both n = 4). At day 7, senescence scores for all DF were slightly elevated from day 3 levels but again there was no significant difference between ND-DF and T2DM-DF (3.20 ± 1.16 vs 2.21 ± 0.85 A.U., *P* = 0.5718, two-way ANOVA, n = 4), and TNF-α had no impact under any conditions (*P* = 0.5196, two-way ANOVA, both n = 4). Senescence-associated β-galacotosidase analysis at day 14 was not performed, as the cells had grown too confluent for individual cells to be visualised.

### TNF-α induces transient changes in ND-DF morphology

**Figure 2:**
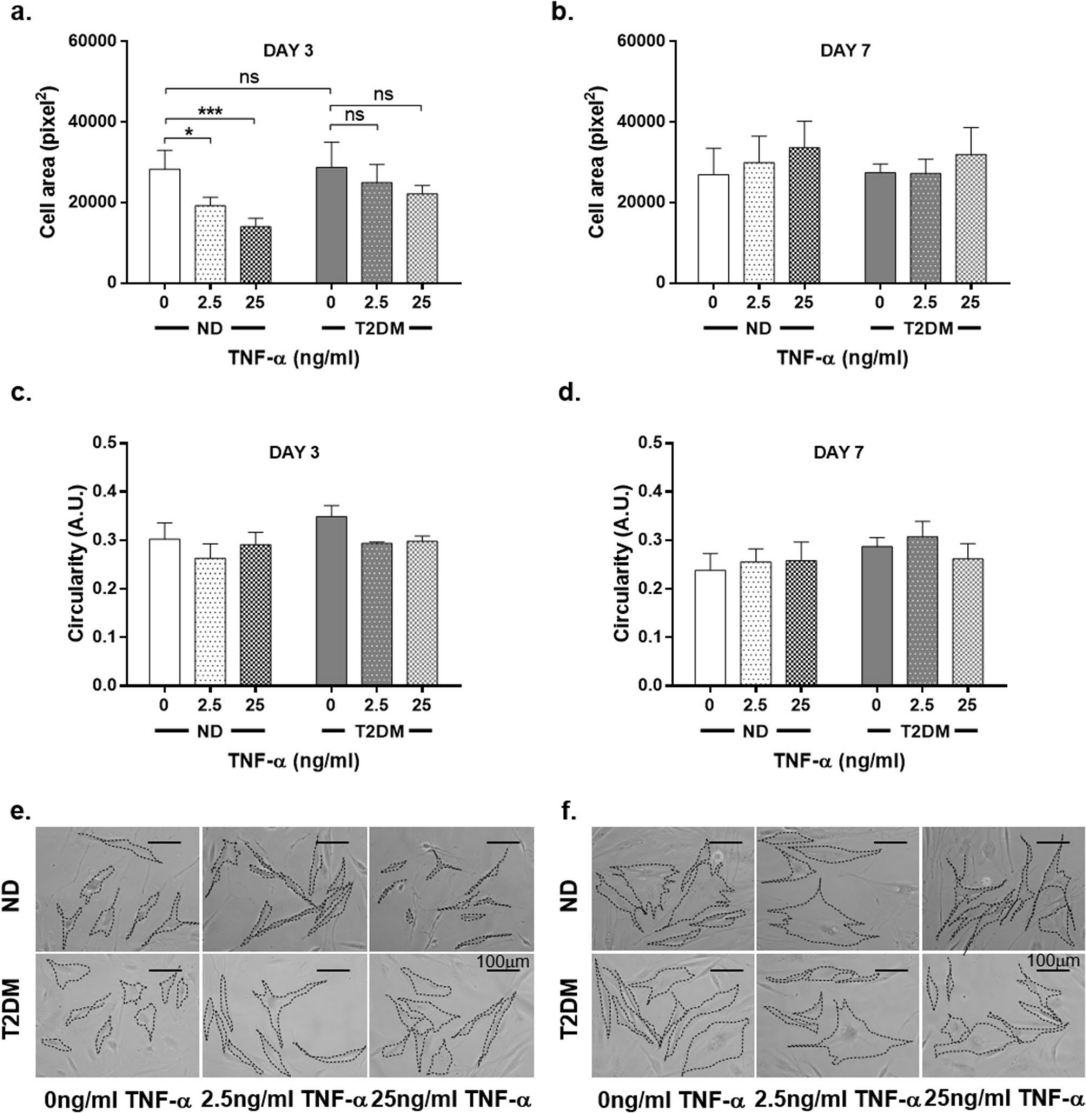
TNF-α has a differential effect on ND-DF and T2DM-DF morphology. The morphology of DF in images taken from the senescence assay were analysed using Image J. (**a**) Mean spread cell area and (**b**) circularity of ND- and T2DM-DF at day 3 post-TNF-α withdrawal (both n = 4 donors). (**c**) Representative images. (**d**) Mean spread cell area and (**e**) circularity of ND-DF and T2DM-DF at day 7 post-TNF-α withdrawal (both n = 4 donors). (**f**) Representative images. Two-way ANOVA with Sidak post-hoc test *P  < 0.05, ***P < 0.001, ns = non-significant.

The morphological characteristics of the cells in the senescence assay were measured to assess whether TNF-α exposure was influencing fibroblast phenotype in vitro. Reductions in cell size indicate quiescence, whereas changes in circularity/cell projections can represent DF activation^31,32^ Three days after withdrawal of TNF-α, there was a significant, concentration-dependent decrease in spread cell area in ND-DF (32.1% reduction in cell area with 2.5 ng/ml, 50.4% reduction with 25 ng/ml TNF-α; *P* = 0.012 for effect of TNF-α, two-way ANOVA n = 4). However, there was no change in circularity suggesting that the cells were simply becoming smaller in size rather than more spindle shaped. In contrast, TNF-α had no impact on T2DM-DF morphology (Fig. 2a,c,e, n = 4). By day 7, the TNF-α-induced reduction in ND-DF spread cell area was lost (Fig. 2b,d,f, n = 4).

### TNF-α inhibits migration in both ND-DF and T2DM-DF

**Figure 3:**
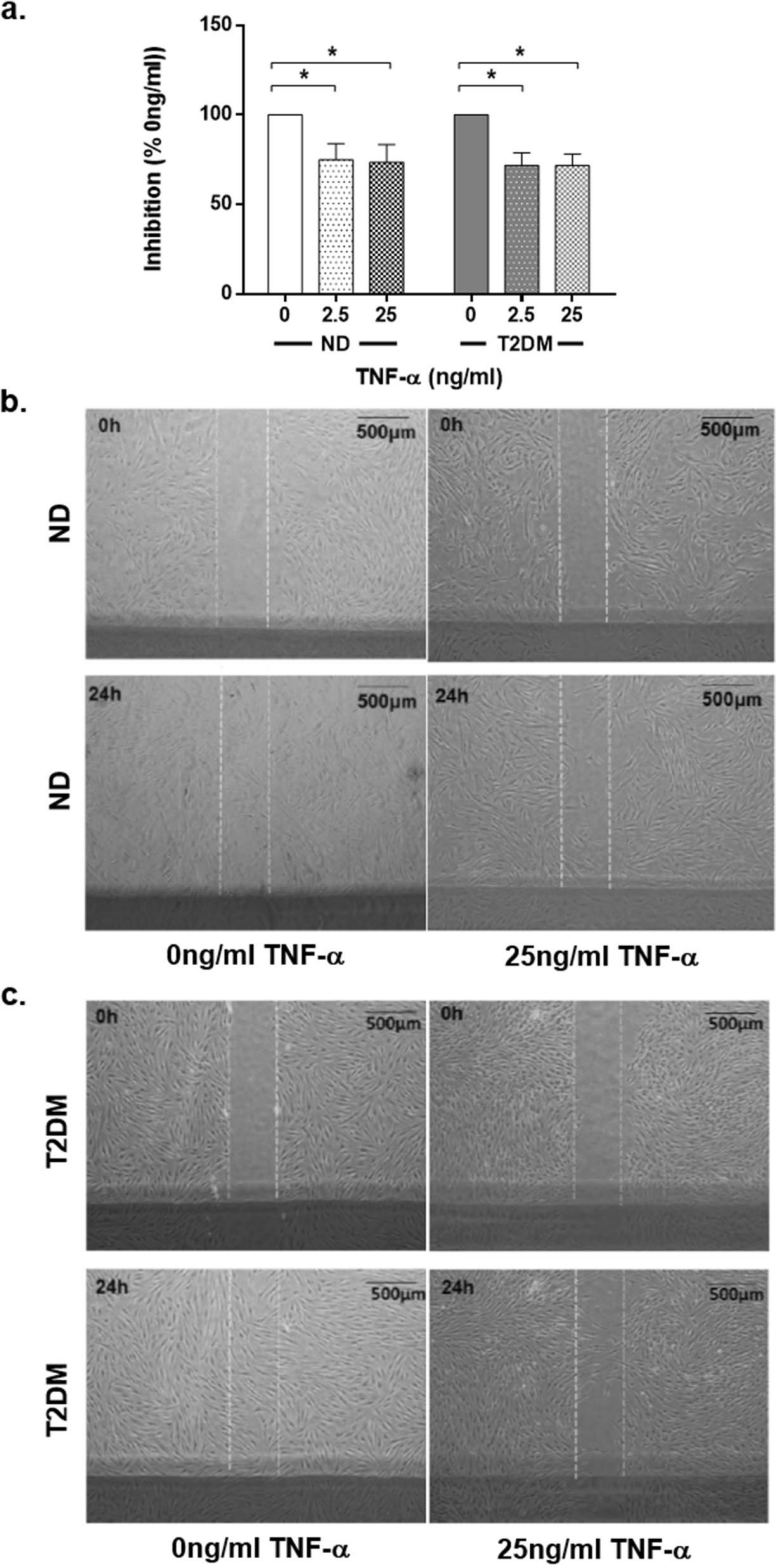
Migratory capacity of ND-DF and T2DM-DF. (**a**) Confluent cells were quiesced for 24 h before being scratched, stimulated ± 0–25 ng/ml TNF-α and imaged. Repeat images were taken of the same wounds 24 h later. The percentage inhibition of TNF-α on migration was calculated by dividing the distance migrated in control cells by the distance migrated in treated cells (both n = 4 donors). (**b**) ND-DF representative images, (**c**) T2DM-DF representative images. Two-way ANOVA with Sidak post-hoc test, *P < 0.05, ns = non-significant.

As wound healing and tissue regeneration requires both cell proliferation and migration to act in concert, we also evaluated the impact of TNF-α on cell migration using the scratch-wound assay. TNF-α significantly inhibited migration by ~ 25% at both 2.5 and 25 ng/ml, and in both ND-DF and T2DM-DF (*P* = 0.0008 for effect of TNF-α, two-way ANOVA, both n = 4; Fig. 3a-c).

### MMP-9 is selectively upregulated by TNF-α in ND-DF and T2DM-DF

**Figure 4:**
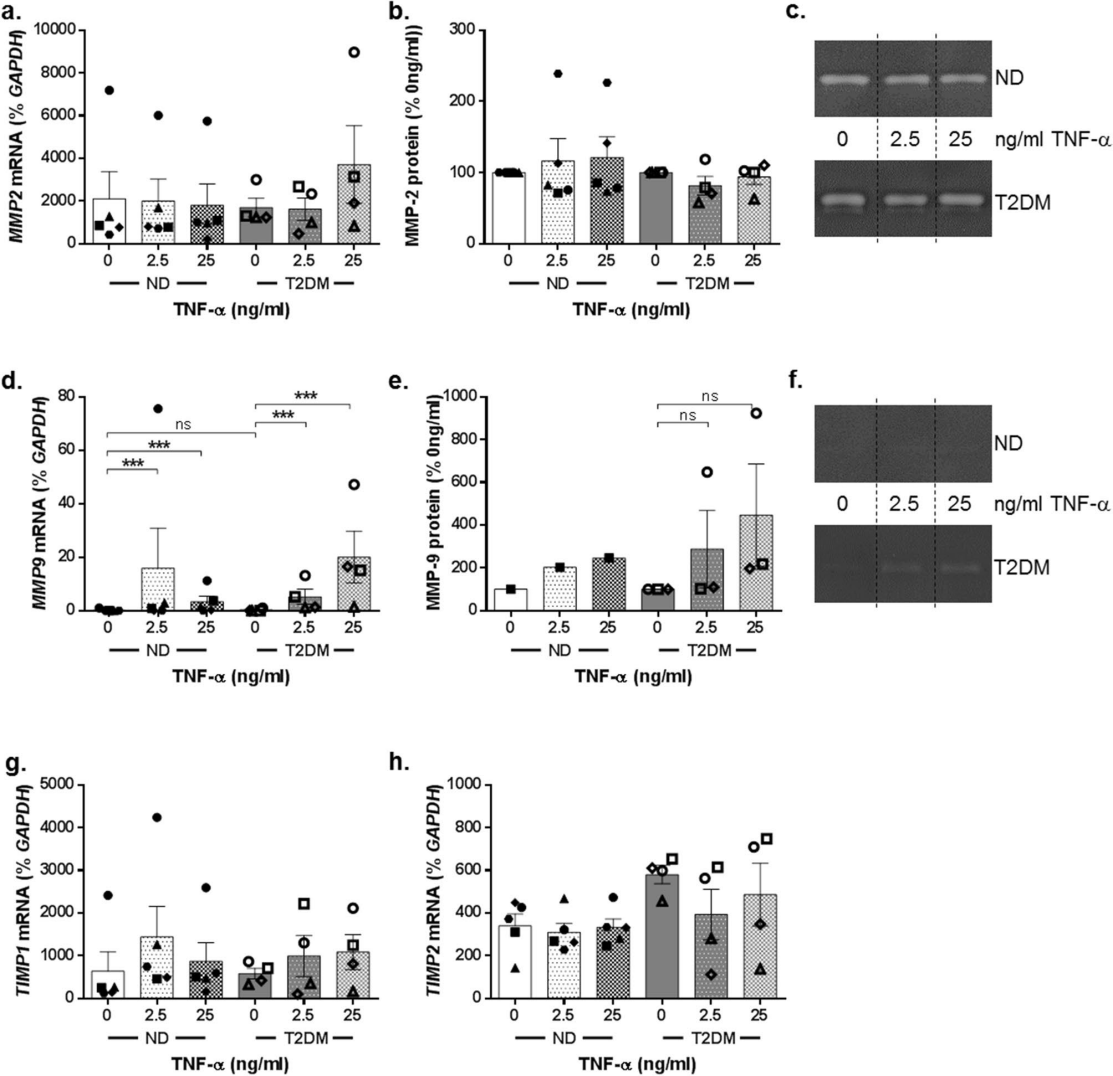
Matrix remodelling in ND-DF and T2DM-DF. (**a**) Cells used in the migration assay were collected and total RNA extracted. *MMP2* gene expression was quantified and expressed relative to *GAPDH* (both n = 4 donors). (**b**) Conditioned media from the migration assays was analysed for MMP-2 secretion using gelatin zymography (both n = 4 donors), (**c**) representative images. (**d**) *MMP9* gene expression and (**e**–**f**) protease activity were analysed using the same method (both n = 4 donors). (**g**) *TIMP1* and (**h**) *TIMP2* gene activity in the same cells (both n = 4 donors). Two-way ANOVA with Sidak post-hoc test ***P < 0.001, ns = non-significant.

The gelatinases MMP-2 and MMP-9 both facilitate fibroblast migration during the wound healing response. *MMP2* gene expression was unaffected by either concentration of TNF-α, or the presence of diabetes (ND-DF n = 5, T2DM-DF n = 4, Fig. 4a) and this was mirrored at the protein level (n = 4, Fig. 4b-c). In contrast *MMP9* gene expression was significantly increased by TNF-α at both 2.5 and 25 ng/ml, and in both ND-DF and T2DM-DF to a comparable degree (*P* < 0.0001 for effect of TNF-α, two-way ANOVA, n = 5 ND-DF and n = 4 T2DM-DF; Fig. 4d). MMP-9 protein was only detected by zymography in one ND-DF line (where it increased in a TNF-α concentration-dependent manner), yet it was readily detected in 3 out of the 4 T2DM-DF, where there was a suggestion that it may increase in a TNF-α concentration dependent manner, though this was not statistically significant (Fig. 4e-f).

Neither *TIMP1* nor *TIMP2* gene expression was affected by either concentration of TNF-α, however T2DM-DF had a 1.7-fold higher basal expression of *TIMP2* than ND-DF (579.8 ± 42.9 vs 340.4 ± 54.8% *GAPDH* respectively; ND-DF n = 5, T2DM-DF n = 4, Fig. 4g-h), although this did not reach statistical significance.

### *TNF-α induces the expression of the pro-inflammatory cytokines CCL2, CXCL1 and SERPINE1 in DF *in vitro

**Figure 5:**
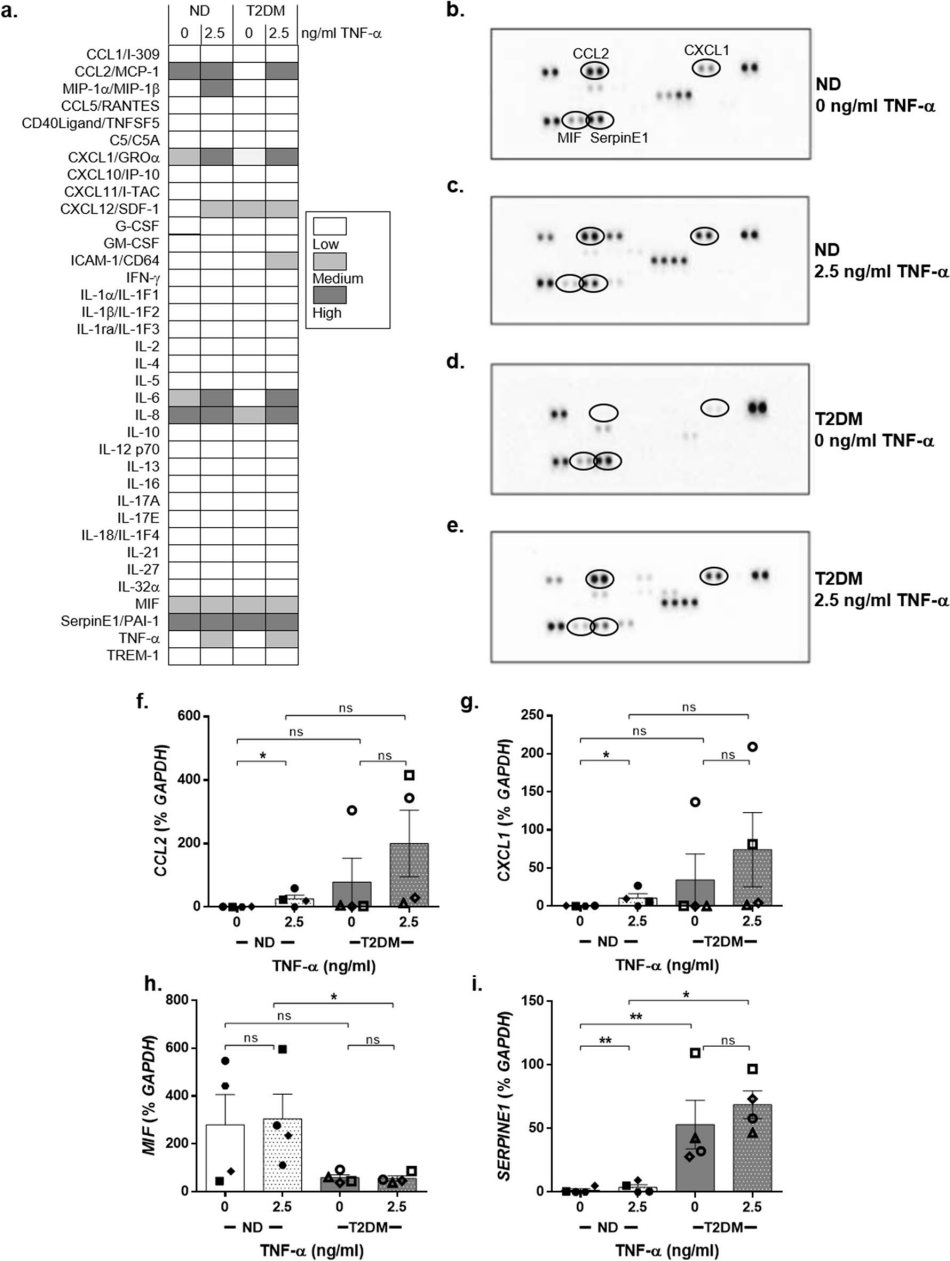
Cytokine secretion in ND-DF and T2DM-DF. The secretion of multiple cytokines was measured in conditioned media from the migration assays using R&D Systems Human Cytokine Array Panel A. (**a**) Heatmap of the cytokines contained within the array and expression in ND and T2DM-DF, both basally and in response to 2.5 ng/ml TNF-α. (**b**) Representative dot blot used in the heatmap analysis for ND-DF under control (basal conditions), (**c**) ND-DF in response to TNF-α, (**d**) T2DM-DF under control (basal conditions) and (**e**) T2DM-DF in response to TNF-α. (**f**) Gene expression of *CCL2*, **(g)** *CXCL1*, (**h**) *MIF* and (**i**) *SERPINE1* was measured in the cells from the migration assays (both n = 4 donors). Two-way ANOVA with Sidak post-hoc test, **P < 0.01, *P < 0.05, ns = non-significant.

T2DM is a hyper-inflammatory condition that impacts on wound healing. Therefore, we sought to assess whether there were any basal differences in the pro-inflammatory cytokine expression profile between ND-DF and T2DM-DF, and whether TNF-α stimulation altered this profile. The Cytokine Profiler Array indicated that 9 pro-inflammatory cytokines were secreted by ND-DF and T2DM-DF (Fig. 5a-e). From these, we chose 4 candidates to validate using RT-PCR, to quantitate differences in their level of production. These were C–C motif ligand 2 (CCL2, also known as monocyte chemoattractant protein 1, MCP-1), C-X-C motif chemokine ligand (CXCL1, also known as fibroblast secretory protein, FSP), macrophage migration inhibitory factor (MIF) and SERPINE1 (also known as plasminogen activator inhibitor 1, PAI-1).

Basal *CCL2* expression was higher in T2DM-DF (78.7 ± 75.3 vs 0.8 ± 0.4% *GAPDH*, n = 4), however this was skewed by one T2DM-DF population with an exceptionally high expression level (*P* = 0.0733, two-way ANOVA, n = 4). TNF-α induced *CCL2* expression in ND-DF by 32-fold over basal unstimulated levels (Fig. 5f, n = 4). *CXCL1* expression followed a similar pattern, whereby basal expression level was higher in T2DM-DF (skewed by the same T2DM-DF population; *P* = 0.2870, two-way ANOVA, n = 4) and was significantly induced by TNF-α in ND-DF only, by 27-fold (Fig. 5g, n = 4).

In contrast, *MIF* expression was significantly reduced in T2DM-DF compared to ND-DF (58.7 ± 12.3 vs 280.4 ± 126.2% *GAPDH*; *P* = 0.0016 for effect of diabetes, two-way ANOVA, n = 4), while TNF-α had no modulatory effect in either (*P* = 0.7303 for effect of TNF-α, two-way ANOVA, n = 4; Fig. 5h). *SERPINE1* expression was significantly higher in T2DM-DF (53.03 ± 19.1 vs. 1.42 ± 1.19% *GAPDH*; *P* = 0.0072 for effect of T2DM, two-way ANOVA, n = 4). Whilst TNF-α induced a significant 2.6-fold increase in *SERPINE1* mRNA in ND-DF, there was no effect of TNF-α in T2DM-DF (*P* = 0.0035 for effect of TNF-α, two-way ANOVA, n = 4; Fig. 5i).

## Discussion

During the early phase of wound repair, there is increasing evidence that DF are a primary source of TNF-α^33^. While there have been no previous studies comparing TNF-α secretion in DF from patients with or without diabetes, we hypothesised that there may be significant differences in the secretion and/or response to TNF-α by DF derived from T2DM skin that may impact on wound healing. We initially compared the basal proliferation, migration and secretion of TNF-α by primary cultures of ND-DF and T2DM-DF (Figs. 1a-c) and found no significant difference. Furthermore, although pre-incubation with TNF-α significantly upregulated its secretion, there was no difference between the two cell types (Fig. 1c), demonstrating any differences observed in response to TNF-α (e.g. proliferation, migration, secretion of cytokines) are not due to underlying differences in the amount of TNF-α the cells may produce.

Therefore, we sought to determine whether their responses to exogenous TNF-α differ. In normal wound healing TNF-α levels increase approximately 12 to 24 h after wounding, returning to basal levels following the proliferative phase^24^, so we exposed cultured ND-DF and T2DM-DF to supra-physiological doses of TNF-α for 3 days, before removing and culturing in the absence of TNF-α for a further 11 days (14 days in total) to mimic this transiency. While TNF-α had no direct effect on cell number at day 3, prior exposure significantly inhibited ND-DF proliferation (Fig. 1d) but did not reduce T2DM-DF proliferation (Fig. 1e) by day 14. In vivo, human DF are not rapidly dividing cells; their primary function is to maintain the homeostasis of the dermis by collagen synthesis and modelling of the ECM^34^. Therefore, following the proliferative phase of wound repair, DF proliferation abates to enter the remodelling phase. Our data suggest T2DM-DF have lost the normal response to the resolution of the inflammatory phase and subsequent reduction in circulating TNF-α which may likely impair the synthesis and remodelling of the ECM.

Since sub-lethal doses of pro-inflammatory cytokines induce senescence, we next measured expression of senescence-associated β-galactosidase following pre-incubation with TNF-α. However, this did not induce senescence in either ND-DF or T2DM-DF, indicating that reduced cell number in ND-DF was not due to permanent withdrawal from the cell cycle (Fig. 1f-i). Reduction in cell number can be indicative of transient cell cycle withdrawal – quiescence – and so we compared the effect of TNF-α on cell morphology. Three days after TNF-α withdrawal there was a significant decrease in spread cell area in ND-DF (Fig. 2a), returning to control levels at day 7. In contrast, TNF-α did not impact T2DM-DF morphology. Collectively, this suggests rather than senescence, TNF-α induces quiescence, by which normal fibroblasts halt proliferation but remain metabolically healthy. While quiescent fibroblasts have reduced size, and are non-proliferating, they have a high metabolic activity^31^. A recent study has reported that when human mesenchymal stem cells quiesce in response to environmental challenges, they have a higher tolerance to stress than proliferating cells, important for ensuring tissue maintenance^35^. The high metabolic activity reported by quiescent fibroblasts is likely due to ECM synthesis, since contact-inhibited fibroblasts secrete more fibronectin, collagen, and laminin than proliferating fibroblasts^31^. In the present study, the shift in ND-DF phenotype (Fig. 2a) may be particularly significant during wound repair, because subsequent to the inflammatory phase, quiescent, non-proliferating fibroblasts are essential for ECM synthesis and tissue homeostasis. It is noteworthy that this was not observed in T2DM-DF, suggesting their normal physiological responses have been lost and may explain the impaired production of granulation tissue in diabetic patients that can result in a non-healing wound.

Fibroblast migration is also key to wound repair. A previous study reported that TGF-α stimulated migration of cultured human DF is inhibited by 20 ng/ml TNF-α by suppressing trans-differentiation into myofibroblasts^36^. In mice, diabetic DF have an impaired migratory ability, migrating 75% less than the normal DF^37^. In contrast to the differential proliferative and quiescent effects (Figs. 1, 2) observed, there was a significant inhibition of ND-DF and T2DM-DF migration in response to TNF-α (Fig. 3), where both responded in a similar manner.

A pro-inflammatory environment can increase MMP expression which has been implicated in impaired wound healing in both T2DM and animal models of diabetic skin^24,38,39^. Furthermore, in a pro-inflammatory environment, increased activity of MMPs has been shown to impair dermal fibroblast migration^15,20,40^. Therefore, we sought to establish whether there was a difference in expression and activity of MMPs and TIMPS between ND-DF and T2DM-DF, or their responses to TNF-α. We observed no difference in MMP-2 expression at either gene or protein level, regardless of diabetic status or the presence of TNF-α (Fig. 4a-c). However, MMP-9 mRNA expression was induced to a similar degree by TNF-α in both cell types (Fig. 4d). Most studies indicate that normal human DF do not express MMP-9, and in keeping with this, protein expression of MMP-9 was detected in only one of the four ND-DF donors, but in three of the four T2DM-DF (Fig. 4e). Chronic diabetic wounds have been reported to have an imbalance of MMP:TIMP ratios, skewed in favour of increased MMPs^9,41^. Indeed, the MMP-9/TIMP-1 ratio in wound fluids correlates with the probability of ulcer healing, highlighting the detrimental effect of MMP-9 in chronic wounds^42^. In the present study, expression of both *TIMP1* and *TIMP2* were unaffected by TNF-α (Fig. 4g-h), although interestingly T2DM-DF had a higher basal level of TIMP2 mRNA than ND-DF (Fig. 4h).

In addition to ECM modulators (MMPs and TIMPs), DF also secrete pro-inflammatory cytokines, so we sought to identify differences between ND-DF and T2DM-DF in their secretion of other pro-inflammatory cytokines, and if these were modulated by TNF-α (Fig. 5a-e). A number were secreted by both ND-DF and T2DM-DF, including CCL2, CXCL1, MIF, SERPINE1/PAI-1, chemokine C-X-C motif ligand 12/ stromal cell-derived factor 1 (CXCL12/SDF-1), interleukin-6 (IL-6) and interleukin-8 (IL-8). Full-skin substitutes and autograft studies have shown DF and keratinocytes secrete IL-6, IL-8, CCL2 and CXCL1, important mediators of wound healing^30^. IL-6 is increased in both human and animal chronic wounds^43–46^ and induced in DF by TNF- α^47^. Our observation that both IL-6 and IL-8 were upregulated by TNF-α (Fig. 5a) concurs with this, confirming DF respond to TNF-α in culture by increasing secretion of pro-inflammatory cytokines.

The expression of CCL2, CXCL1, MIF and SERPINE1/PAI-1 were further validated by qRT-PCR (Fig. 5f-i). DF secrete CCL2 to accelerate leukocyte homing and promote endothelial activation in inflammation^48^. Likewise, CXCL1 promotes neutrophil infiltration. In the present study, expression of CCL2 and CXCL1 was highly variable in T2DM-DF with neither modulated by TNF-α, while in contrast TNF-α increased expression of both CCL2 and CXCL1 in ND-DF (Fig. 5f-g). Reduced sensitivity to TNF-α by T2DM-DF may reflect their higher basal expression of these cytokines. MIF has a role in the insulin biosynthesis pathway^49^, although its exact role in wound healing is still a matter of debate^50,51^. Similarly, fluctuations in MIF concentrations in chronic venous ulcers lack consensus^52,53^. In the current study, TNF-α had no effect on MIF expression in either cell type, but expression was significantly lower in T2DM-DF (Fig. 5h). One function of MIF is to stimulate the chemotactic migration of endothelial progenitor cells (EPCs) and promote revascularisation^53^. Therefore, reduced expression of MIF in T2DM-DF may be one factor in impaired angiogenesis in diabetic wounds. SERPINE1 has pro-inflammatory effects including macrophage activation, and a well-described role in fibrosis^54^. TNF-α is a strong agonist for SERPINE1 expression and may contribute to elevated plasma levels in obesity, indeed high levels are considered to be biomarkers for T2DM^55^. We observed significantly higher SERPINE1 expression in T2DM-DF, and whilst TNF-α significantly increased expression in ND-DF, it did not modulate expression in T2DM-DF (Fig. 5i), again suggesting T2DM-DF have lost their normal responses to TNF-α. The significantly higher levels expressed by T2DM-DF may contribute to dysregulated maintenance of the provisional fibrin matrix by inhibiting fibrinolysis in the early stages of wound healing, thus impeding the resolution of inflammation.

The persistence of hyperglycemia-associated epigenetic patterns sustains and drives progression of disease phenotypes, despite removal of the glycemic environment^56^. The role of epigenetic metabolic memory in establishing a diabetic phenotype has been demonstrated in a number of cell types, including DF derived from chronic, non-healing diabetic foot ulcers^57^ and smooth muscle cells from T2DM patients^58^. Our data highlight significant differences between T2DM-DF and ND-DF supporting the view that epigenetic-related metabolic memory in human DF may be the basis for the divergence in phenotypes associated with poor wound healing outcomes in T2DM patients. DF are important mediators of crosstalk between multiple cell types vital for normal wound healing, therefore, understanding their role in both the initiation and maintenance of the diabetic non-healing wound environment is paramount in the drive to identify new therapeutic targets for the treatment of chronic, non-healing diabetic foot ulcers.

## Methods

### Dermal fibroblast isolation

DF were isolated from human skin provided by Ethical Tissue, University of Bradford. The use of human skin for this project was granted by the Yorkshire & the Humber—Leeds East Ethics Committee (reference numbers 17/YH/0086 and 07/H1306/98 + 5) and all donors gave informed consent. All methods were performed in accordance to the relevant guidelines and regulations. DF were isolated using an explant technique as previously described^59^. Briefly underlying fat was removed, before cutting skin into 1 cm^2^ pieces, washing in phosphate buffer saline (PBS) containing penicillin/streptomycin (100U/ml/100 μg/ml) and amphotericin B (250 μl/ml), and digesting overnight at 4C in 0.1% dispase in PBS. The epidermis was removed and pieces of dermis washed in growth media (Dulbecco’s Modified Eagle’s Medium (DMEM) supplemented with 10% foetal bovine serum (FBS) l-glutamine (10 mM) and penicillin, streptomycin and amphotericin B (100 U/ml, 100 μg/ml, 250 μl/ml respectively), before transferring to a T75 tissue culture flask containing fresh media and incubating at 37 °C in 5% CO_2_ to allow DF explant, which took 5–10 days. Once confluent cells were serially passaged at a ratio of 1:3. Patients without diabetes (ND) demographics were 100% female, age range 36–67 years. Patients with diabetes (T2DM) were 50% female, age range 52–66 years. All experiments were performed on cells at passage 3–6.

### ELISA

DF were quiesced for 24 h and then treated with TNF-α (0–2.5 ng/ml) in DMEM + 10% FBS for 24 h. Conditioned medium was collected and the concentration of TNF-α quantified using a Human TNF-α Quantikine ELISA (R&D Systems) according to manufacturer’s instructions.

### Proliferation

Basal DF proliferation rates were assessed by cell counting. DF were plated in triplicate at a density of 20,000 cells per well in 24-well plates. Cells were quiesced in DMEM containing 0% serum (SF medium) for 24 h and then treated with DMEM + 10% FBS for up to 7 days. Viable and non-viable cells were counted using trypan blue and a haemocytometer, and media replenished, on days 0, 3, 5 and 7.

To assess the impact of TNF-α on proliferation, DF were seeded in 96-well plates in quadruplicate at a density of 2,000 cells per well, quiesced in SF DMEM for 24 h and then treated with TNF-α (0–250 ng/ml) in DMEM + 10% FBS for 72 h. Medium was removed and replaced with DMEM + 10% FBS only for up to 14 days, and replenished every 3 days. DF proliferation was quantified using the CyQUANT fluorescence-based assay according to manufacturers’ instructions (Invitrogen). Fluorescence was measured at excitation 508 nm and emission 527 nm. Cell number was interpolated from a standard curve of 1–5000 cells at days 3, 7 and 14.

### Senescence

DF were seeded in 6-well plates at a density of 7000 cells per well and treated as described for the proliferation assay. At days 3, 7 and 14, cells were fixed, and senescence approximated using the senescence-associated β-galactosidase kit (Cell Signaling Technology) according to manufacturers’ instructions. After staining, 5 random fields of view (× 100 magnification) were captured from each treatment well for each patient, and a senescence score calculated from the ratio of positively stained senescent cells (blue) and the total number of cells present in the field as previously described^60^.

### Morphological measurements

Morphological characteristics of the cells were captured from the images generated from the senescence staining. Fifty random cells per patient per treatment were traced using Image J, and the spread cell area and circularity quantified^58^.

### Scratch wound migration

Confluent DF were quiesced in SF medium for 24 h. A 0.8 mm wide linear scratch was made in triplicate monolayers and dislodged cells removed by washing in PBS^61^. DF were incubated in DMEM + 10% FBS with 0–25 ng/ml TNF-α for 24 h. Images were captured at × 40 magnification at 0 and 24 h. The migration distance between the wound edges was measured at 6 identical points in triplicate wells and the average taken. For TNF-α experiments, data is expressed as the percentage inhibition of migration compared to control (0 ng/ml) wells.

### Gene expression

Following 24 h imaging of the migration assays, total RNA was extracted from DF using RNeasy mini kit (Qiagen) and reverse transcribed using the High-Capacity cDNA Reverse Transcription Kit (Applied Biosystems) according to manufacturers’ instructions. Gene expression of *CCL2*, *CXCL1, GAPDH*, *MIF*, *MMP2*, *MMP9*, *TIMP1*, *TIMP2*, and *SERPINE1* was measured in triplicate using specific TaqMan assays and expressed as a proportion of *GAPDH* using the formula 2^−ΔCt^.

### Gelatin zymography

Confluent DF were quiesced in SF medium for 24 h and treated with 2.5–25 ng/ml TNF-α for 48 h. Conditioned medium was collected, centrifuged at 600 g for 6 min to remove cell debris and snap frozen until needed for zymographical analysis. Conditioned medium was diluted (1:40) with non-reducing sample buffer and electrophoresed in an 10% SDS-gel containing 1.5 mg/ml gelatin for 110 min at 120 V. SDS was removed by washing in 2.5% Triton X-100 solution for 1 h and the gels placed in incubation buffer (50 mM Tris–HCl, 10 mM CaCl_2_, 0.05% Brij, pH7.4) for 18 h (MMP-2 activity) or 24 h (MMP-9). Gels were stained with 0.1% Coomassie blue for 20 min and gelatinase activity visualised as clear bands of lysis on a dark background. MMP-2 and MMP-9 were measured using densitometry (Image J) and normalised to control (0 ng/ml) values^62^. Conditioned media from ND-DF treated with 100 nM phorbol 12-myristate 13-acetate (PMA) for 24 h was used as a marker for MMP-2 and MMP-9.

### Cytokine profiler array

Conditioned medium collected from the migration assays was used to evaluate a number of inflammatory cytokines using the Human Cytokine Array Panel A (R&D Biosystems) as per manufacturers’ instructions. The conditioned medium was centrifuged to remove any cellular debris and 1 ml was diluted with 0.5 ml of assay buffer (see https://www.rndsystems.com/products/proteome-profiler-human-cytokine-array-kit_ary005b for full methodology). Following completion, integrated density of the dots was calculated. Briefly, the mean densitometry of duplicate dots was measured using Image J. The reciprocal was calculated which was then corrected for the value of the background densitometry. Then, the integrated density of each pair of cytokines was expressed as a proportion of the integrated density of the reference spots that were included on all membranes. Expression was stratified into low (< 0.1 A.U.), medium (0.1–2 A.U.) or high (> 2 A.U.).

### Statistical analysis

Data are presented as mean ± standard error. Normalised data was transformed using the formula Y = log[Y]. *n* refers to the number of patient donors used in each experiment. All data was analysed using two-way ANOVA with Sidak post-hoc test. Alpha = 0.05 and *P* < 0.05 was considered statistically significant.

## Supplementary Information

Supplementary Information.

## Data Availability

All data are available upon request.
