# Supplementary material for: Dermal fibroblasts cultured from donors with type 2 diabetes mellitus retain an epigenetic memory associated with poor wound healing responses
Source: Sci Rep. 2021 Jan 14;11:1474. doi: 10.1038/s41598-020-80072-z (PMC7809350; doi:10.1038/s41598-020-80072-z)

**SUPPLEMENTARY INFORMATION**

**Dermal fibroblasts cultured from donors with type 2 diabetes mellitus retain an epigenetic memory associated with poor wound healing responses**

Aaiad H. A. Al-Rikabi^1^,

Desmond J. Tobin^2^**,**

Kirsten Riches-Suman^1^,

M. Julie Thornton^1^*

^1^ Centre for Skin Sciences, School of Chemistry and Biosciences, Faculty of Life Sciences, University of Bradford, Bradford, UK, BD7 1DP

^2^ The Charles Institute for Dermatology, School of Medicine, University College Dublin, Dublin, Ireland

* Corresponding author: Dr M. Julie Thornton, Centre for Skin Sciences, University of Bradford, UK, BD7 1DP. Email: m.j.thornton@bradford.ac.uk. Tel: (+44)1274 235517

*Viability assay*

DF were pated at a density of 20,000 cells per well in 24 well plates and quiesced for 24 h. Cells were treated with DMEM+10% FBS for up to 7 days. Cell counts were taken on days 3, 5 and 7. Media was collected and cells were trypsinised. Both media and cells were pelleted and resuspended in 50 μl DMEM and mixed 1:1 with Trypan Blue. The percentage of blue (dead) cells for each treatment was calculated for both the media and cells for each condition.

**Supplementary Figure 1: TNF-α has no impact on cell viability.** ND-DF were plated at a density of 20,000 cells per well, quiesced and treated with DMEM+10% FBS for up to 7 days. At days 3, 5 and 7 the number of dead / floating cells in each well were calculated as a proportion of the total number of healthy cells using Trypan Blue staining (n=2).

**Supplementary Figure 2: Full length zymograms.** Full electropherograms for the data presented in Figure 4. (a) MMP-2 in ND-DF, (b) MMP-2 in T2DM-DF, (c) MMP-9 in ND-DF, (d) MMP-9 in T2DM-DF.


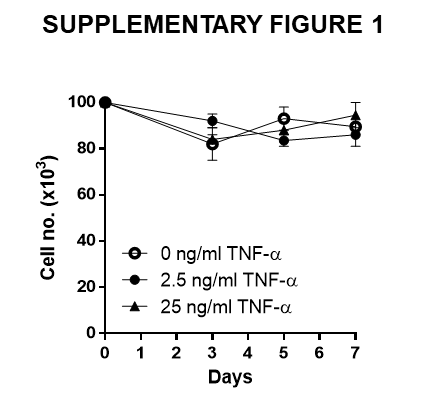


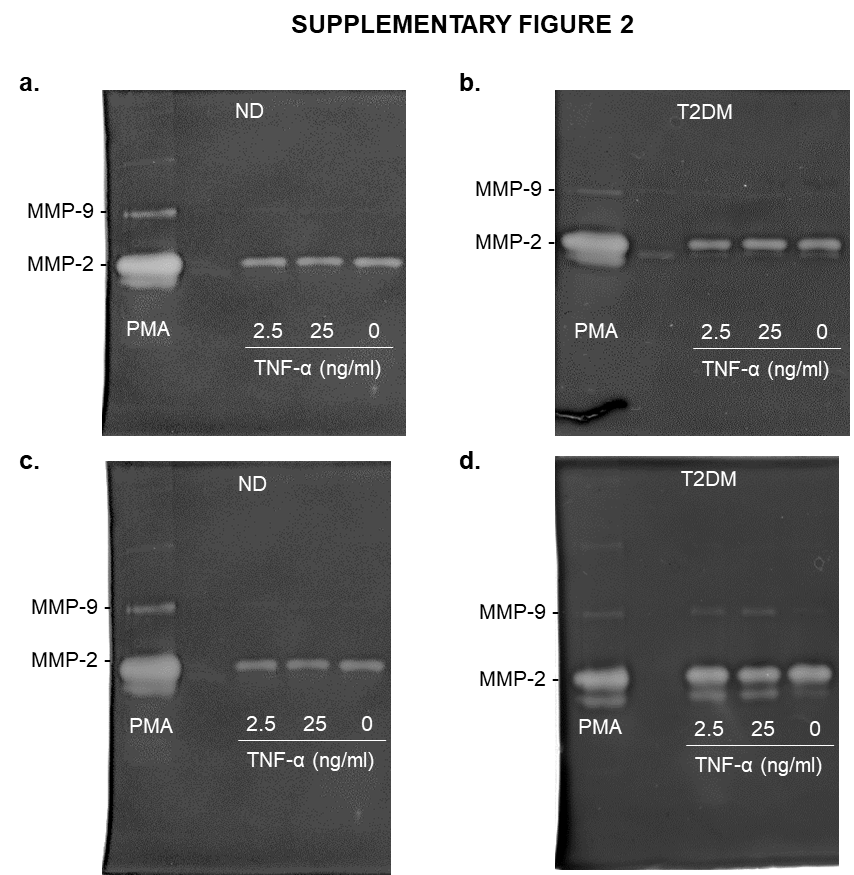

Supplement: Supplementary file 1 — Supplementary Information. [file 41598_2020_80072_MOESM1_ESM.docx]
